# Adverse Childhood Experiences and Adult Mental Health Outcomes

**DOI:** 10.1001/jamapsychiatry.2024.0039

**Published:** 2024-03-06

**Authors:** Hilda Björk Daníelsdóttir, Thor Aspelund, Qing Shen, Thorhildur Halldorsdottir, Jóhanna Jakobsdóttir, Huan Song, Donghao Lu, Ralf Kuja-Halkola, Henrik Larsson, Katja Fall, Patrik K. E. Magnusson, Fang Fang, Jacob Bergstedt, Unnur Anna Valdimarsdóttir

**Affiliations:** 1 Centre of Public Health Sciences, Faculty of Medicine, University of Iceland, Reykjavík, Iceland; 2 Institute of Environmental Medicine, Karolinska Institutet, Stockholm, Sweden; 3 Clinical Research Center for Mental Disorders, Shanghai Pudong New Area Mental Health Center, Tongji University School of Medicine, Shanghai, China; 4 Institute for Advanced Study, Tongji University, Shanghai, China; 5 Department of Psychology, Reykjavík University, Reykjavík, Iceland; 6 West China Biomedical Big Data Center, West China Hospital, Sichuan University, Chengdu, China; 7 Department of Medical Epidemiology and Biostatistics, Karolinska Institutet, Stockholm, Sweden; 8 Clinical Epidemiology and Biostatistics, School of Medical Sciences, Örebro University, Örebro, Sweden; 9 Department of Epidemiology, Harvard TH Chan School of Public Health, Boston, Massachusetts

## Abstract

**Importance:**

Exposure to adverse childhood experiences (ACEs) has consistently been associated with multiple negative mental health outcomes extending into adulthood. However, given that ACEs and psychiatric disorders cluster within families, it remains to be comprehensively assessed to what extent familial confounding contributes to associations between ACEs and clinically confirmed adult psychiatric disorders.

**Objective:**

To investigate whether associations between ACEs and adult mental health outcomes remain after adjusting for familial (genetic and environmental) confounding.

**Design, Setting, and Participants:**

This Swedish twin cohort study used a discordant twin pair design based on monozygotic (MZ) and dizygotic (DZ) twins. A total of 25 252 adult twins (aged 18-47 years) from the Swedish Twin Registry born between 1959 and 1998 were followed up from age 19 years until 2016, with a maximum follow-up time of 39 years. Data were analyzed from April 2022 to November 2023.

**Exposures:**

A total of 7 ACEs, including family violence, emotional abuse or neglect, physical neglect, physical abuse, sexual abuse, rape, and hate crime, were assessed with items from the Life Stressor Checklist-Revised in a web-based survey.

**Main Outcomes and Measures:**

Adult (ages >18 years) clinical diagnosis of psychiatric disorders (ie, depressive, anxiety, alcohol or drug misuse, or stress-related disorders) were obtained from the Swedish National Patient Register.

**Results:**

Of 25 252 twins included in the study (15 038 female [59.6%]; mean [SD] age at ACE assessment, 29.9 [8.7] years), 9751 individuals (38.6%) reported exposure to at least 1 ACE. A greater number of ACEs was associated with increased odds of any psychiatric disorder in the full cohort (odds ratio [OR] per additional ACE, 1.52; 95% CI, 1.48-1.57). The association remained but ORs per additional ACE were attenuated in DZ (1.29; 95% CI, 1.14-1.47) and MZ (1.20; 95% CI, 1.02-1.40) twin pairs. Individuals who were exposed to sexual abuse compared with those who were not exposed had increased odds of any clinically confirmed psychiatric disorder in all comparisons: full cohort (OR, 3.09; 95% CI, 2.68-3.56), DZ twin pairs (OR, 2.10; 95% CI, 1.33-3.32), and MZ twin pairs (1.80; 95% CI, 1.04-3.11).

**Conclusions and relevance:**

This study found that associations between ACEs and adult mental health outcomes remained after controlling for shared genetic and environmental factors, which was particularly evident after multiple ACEs or sexual abuse. These findings suggest that targeted interventions may be associated with reduced risks of future psychopathology.

## Key Points

**Question:**

Are adverse childhood experiences (ACEs) associated with poor mental health in adulthood after adjustment for familial confounding due to shared genetic and environmental factors?

**Findings:**

In this cohort study using twin data, there were associations between ACEs and adult mental health outcomes in dizygotic and monozygotic twin pairs, while odds ratios were attenuated compared with the full cohort. Twins who were exposed to ACEs compared with co-twins who were not exposed had increased odds of clinically confirmed adult psychiatric disorders, particularly after sexual abuse or multiple ACEs.

**Meaning:**

These findings support an association between ACEs and poor mental health in adulthood, notwithstanding evidence for familial confounding from shared genetic and environmental factors.

## Teaser

This cohort study investigates the association of adverse childhood experiences with adult psychiatric disorders after adjusting for familial confounding.

## Introduction

Adverse childhood experiences (ACEs) are common, affecting the lives of millions of children and adolescents across the world.^1,2,3,4,5^ ACEs represent exposures to severe stressors, such as emotional, physical, and sexual abuse, as well as growing up in dysfunctional home environments.^6^ A large body of evidence has shown that children exposed to ACEs have increased risks of an array of adverse mental and physical health outcomes throughout life.^7,8,9,10^

Studies have consistently reported associations between ACEs and an increased risk of psychiatric disorders in adulthood, such as posttraumatic stress disorder (PTSD),^11,12^ depression,^13,14,15^ anxiety,^16^ and substance abuse.^17,18^ However, given that ACEs and psychiatric disorders cluster within families,^19,20,21^ few studies examining associations of ACE exposure with subsequent psychiatric disorders have been equipped to disentangle these associations from potential genetic confounding and other risk factors shared by family members. Indeed, twin studies show that psychiatric disorders are moderately heritable, with 40% to 60% of individual differences in, for example, depression, anxiety, and PTSD attributable to genetic factors.^22^ In addition, twin and family studies suggest that liability for ACEs is also partly attributable to genetic factors,^23,24,25^ some of which may overlap with genetic factors that predispose individuals to adverse mental health outcomes.^26^ Previously reported associations between ACEs and psychiatric disorders may therefore, at least partly, reflect genetic confounding. Moreover, adjusting for environmental risk factors that contribute to familial confounding is equally important given that aspects of the family environment, such as parenting style^27^ and socioeconomic disadvantage,^21^ may similarly confound the association between ACEs and psychiatric disorders.

The discordant twin pair design provides a unique opportunity to adjust for unmeasured genetic and early environmental confounders.^28,29^ A handful of previous studies have used this methodology to examine the ACE and mental health association, but these are limited by addressing only specific types of ACEs or selected mental health outcomes, including alcohol dependence, personality disorders, and attention-deficit/hyperactivity disorder (ADHD).^30,31,32,33,34,35,36^ In addition, these studies were based on small samples, cross-sectional designs, or the use of self-reported measures of mental health. To our knowledge, evidence on how ACEs are associated with clinically confirmed psychiatric disorders with adjustment for familial confounding is completely lacking. Therefore, leveraging a nationwide sample of Swedish twins, we aimed to assess associations between ACEs and prospectively ascertained clinical diagnoses of common adult psychiatric disorders while adjusting for familial confounding. To capture a broader range of psychiatric morbidities, we also examined associations between ACEs and self-reported depressive symptoms.

## Methods

An ethical permit was granted for this cohort study using twin data by the regional ethical review board in Stockholm, Sweden, and all participants gave written informed consent for participation. The study followed the Strengthening the Reporting of Observational Studies in Epidemiology (STROBE) reporting guideline.

### Participants

**Figure 1.**
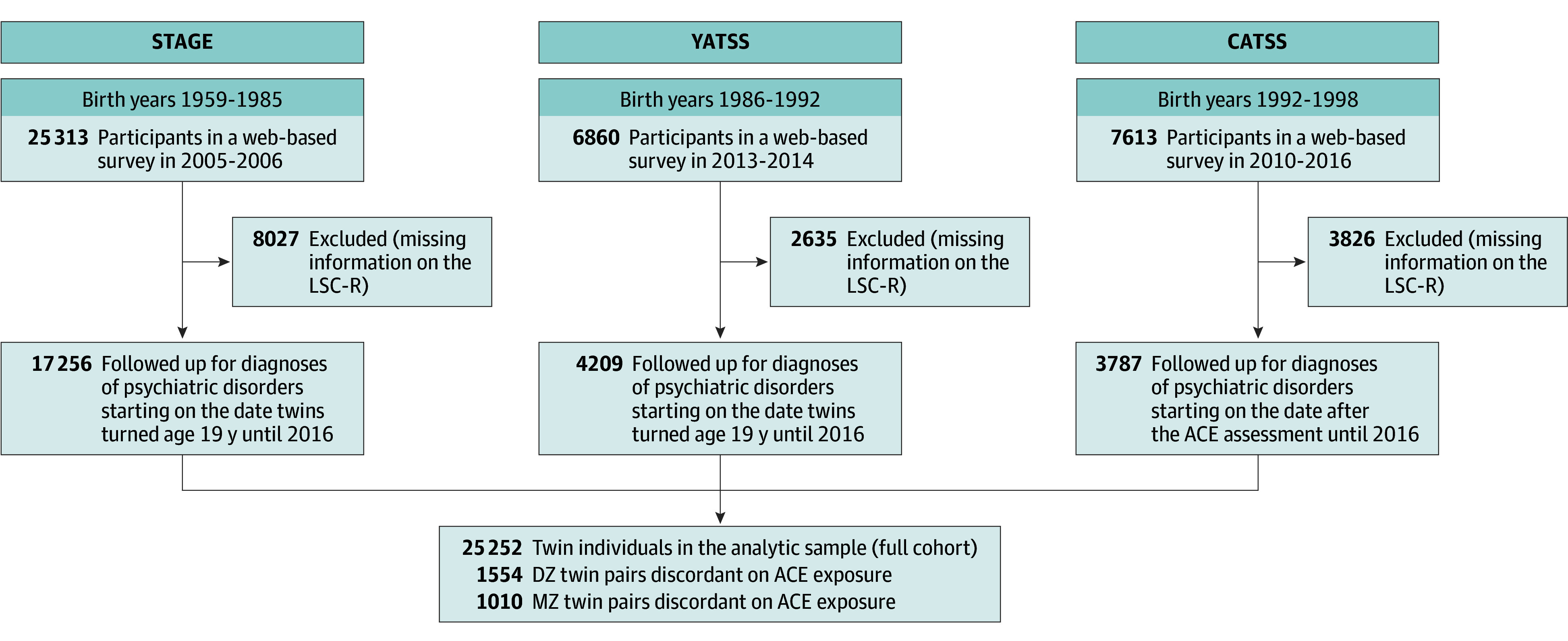
Flowchart of Sample Selection ACE indicates adverse childhood experience; CATSS, Child and Adolescent Twin Study in Sweden; LSC-R, Life Stressor Checklist-Revised; STAGE, Study of Twin Adults: Genes and Environment; YATSS, Young Adult Twins in Sweden Study.

We used data from 3 birth cohorts from the nationwide Swedish Twin Registry. Namely, we used the Study of Twin Adults: Genes and Environment (STAGE) comprising 25 313 twins (born 1959-1985) surveyed 2005 to 2006,^37,38^ the Young Adult Twins in Sweden Study (YATSS) comprising 6860 twins (born 1986-1992) surveyed 2013 to 2014,^39^ and the ongoing Child and Adolescent Twin Study in Sweden (CATSS) comprising 7613 twins (born 1992-1998)^40^ surveyed in relation to their 18th birthday between 2010 and 2016.^40^ All twins responded to web-based surveys, including assessments of ACEs and depressive symptoms, with response rates of 60.0%, 42.0%, and 69.0%, respectively, in the 3 cohorts.^39^ Cohorts were additionally linked to nationwide health registers. In this study, we had access to data on ACEs from 25 252 twins (Figure 1).

### Measures

#### Adverse Childhood Experiences

ACEs were measured with items adapted from the Life Stressor Checklist-Revised (LSC-R).^41,42^ For this study, 7 yes or no questions were used to assess exposure to the following ACEs: emotional neglect or abuse, physical neglect, physical abuse, sexual abuse, rape, hate crime, and witnessing family violence (eTable 1 in Supplement 1). The overall LSC-R has demonstrated good reliability (interrater reliability and test-retest reliability) and validity (content validity and construct validity) to measure trauma exposure and other negative life experiences.^41,42^ Specific items used in this study have acceptable test-retest reliability (κ range, 0.56-0.66).^42^ Follow-up questions were used to assess whether the 7 LSC-R items were endorsed before age 19 years. We examined exposure to the 7 types of ACEs individually (yes or no) and calculated an ACE total score ranging from 0 to 7, which we also categorized into 0, 1, 2, or 3 or more ACEs.

#### Clinical Diagnosis of Psychiatric Disorders

Using Swedish personal identification numbers, the Swedish Twin Registry was linked to the Swedish National Patient Register, established in 1964 with nationwide coverage for inpatient care since 1987 and outpatient specialized care since 2001.^43^ Through this resource, we identified all individuals with any inpatient or outpatient hospital visit that resulted in a diagnosis of a depressive disorder, anxiety disorder, alcohol or drug misuse disorder, or stress-related disorder after their 19th birthday (Figure 1). Participants were followed up in the National Patient Register from age 19 years until the end of 2016, resulting in a follow-up range of 13 to 39 years in STAGE, 6 to 13 years in YATSS, and 0 to 5 years in CATSS. Diagnoses were ascertained in accordance with the Swedish revisions of the *International Classification of Diseases, Eighth Revision* (*ICD-8*), *International Classification of Diseases, Ninth Revision* (*ICD-9*), or *International Statistical Classification of Diseases and Related Health Problems, Tenth Revision* (*ICD-10*) (eTable 2 in Supplement 1).

#### Symptoms of Depression

Symptoms of depression during the past week were measured with the 11-item shortened version of the Center for Epidemiologic Studies Depression Scale (CES-D).^44^ All items were answered on a 4-point scale and then summed to create a total score ranging from 0 to 33, with a higher score indicating higher levels of depressive symptoms.

### Statistical Analysis

Descriptive statistics (means and frequencies) of age at survey, sex, zygosity, and mental health outcomes were summarized for the full analytic sample and by number of ACEs. We calculated the heritability (*h*^2^) of the ACE total score (ie, the proportion of variance attributed to additive genetic factors) with a univariate twin model using the OpenMx package in R statistical software version 4.3.1 (R Project for Statistical Computing).^45^

We used generalized estimating equation analyses with cluster-robust standard errors (drgee package in R)^46^ to examine the association between ACEs and any adult psychiatric disorder and subcategories of these disorders. First, we examined whether ACEs were associated with adult psychiatric disorders in the full cohort (ie, not in twin pairs). Given that outcomes were binary, we estimated odds ratios (ORs) and adjusted the analysis for sex and age (continuous) at the time of the survey. To account for the lack of independence of twin data, we used generalized estimating equation analyses with cluster robust standard errors (ie, using sandwich estimation on twin pair identification number).^46^ We ran all models with the ACE score as a continuous variable (range, 0-7), binary variable (exposed to any ACE vs not exposed), and categorical variable (categories of 0, 1, 2, or ≥3 ACEs). We also carried out stratified analyses of these associations by sex. In addition, we ran separate models for each of the 7 types of ACEs.

Second, discordant twin pair analysis was used to estimate the association between ACEs and psychiatric disorders while controlling for familial (ie, genetic and environmental) factors shared in the twin pair. To do this, we fitted models conditional on twin pairs using generalized estimating equation analyses^46^ separately in monozygotic (MZ) and dizygotic (DZ) twin pairs. These models tested whether a twin exposed to greater levels of ACEs had greater odds of psychiatric disorders compared with their co-twin who had fewer reported ACEs or was not exposed.^29^ Confounding by sex and age was intrinsically adjusted for by the twin design, but given that same- and opposite-sex DZ twin pairs were included, we adjusted for sex in discordant DZ twin pair analyses.

We repeated all cohort-level and discordant twin pair models on self-reported depressive symptoms using generalized estimating equation analyses with identity link. The total score of depressive symptoms was log transformed (log1p function in R), and we obtained estimates as the percentage increase in depressive symptoms for every 1-unit increase in number of ACEs.

#### Sensitivity Analysis

First, to examine whether associations varied by birth cohort, we conducted analyses excluding CATSS given that it is the youngest birth cohort with the shortest follow-up time. We also ran analyses in the youngest 2 birth cohorts (YATSS and CATSS) and the oldest cohort (STAGE) separately, to assess potential modification by birth cohort. Second, because the National Patient Register includes only information related to inpatient or specialized outpatient care, we ran a sensitivity analysis using information from the Swedish Prescribed Drug Register,^47^ using dispensed antidepressants or anxiolytics (Anatomical Therapeutic Chemical codes N06A and N05B, respectively) as an indication of milder psychiatric disorders not requiring specialized care. Third, to inform risks of reverse causation, we carried out sensitivity analyses in which we excluded twins with any diagnosis of psychiatric disorders before age 19 years (eFigure 1 in Supplement 1). Fourth, to perform analyses in which psychiatric disorders were prospectively ascertained after administration of the ACE questionnaire, we restricted the follow-up time of all 3 cohorts to the date after the survey of ACEs and excluded twins who received a diagnosis of any psychiatric disorder before answering the survey (eFigure 2 in Supplement 1).

## Results

Among 25 252 twins (15 038 female [59.6%]; mean[SD] age at ACE assessment, 29.9 [8.7] years), 9751 participants (38.6%) reported exposure to at least 1 ACE. A total of 2046 individuals (8.1%) reported 3 or more ACEs, while 15 501 individuals (61.4%) reported 0 ACEs (Table 1). A univariate twin model showed that variation in exposure to number of ACEs was accounted for by additive genetic factors (34.6%; 95% CI, 25.2%-44.0%), shared environmental factors (22.1%; 95% CI, 14.3%-29.9%), and nonshared environmental factors (43.3%; 95% CI, 40.1%-46.5%).

**Table 1.** Descriptive Characteristics of Study Cohort

Characteristic	Participants, No. (%)				
	Overall (N = 25 252)	0 ACEs (n = 15 501)	1 ACE (n = 5531)	2 ACEs (n = 2174)	≥3 ACEs (n = 2046)
Age at survey, mean (SD), y	29.9 (8.65)	29.9 (8.58)	29.6 (8.71)	30.2 (8.93)	30.6 (8.69)
Sex					
Female	15 038 (59.6)	8914 (57.5)	3319 (60.0)	1385 (63.7)	1420 (69.4)
Male	10 214 (40.4)	6587 (42.5)	2212 (40.0)	789 (36.3)	626 (30.6)
Zygosity					
MZ	9586 (38.0)	6016 (38.8)	2023 (36.6)	806 (37.1)	741 (36.2)
DZ same sex	7337 (29.1)	4504 (29.1)	1639 (29.6)	630 (29.0)	564 (27.6)
DZ opposite sex	7679 (30.4)	4617 (29.8)	1713 (31.0)	675 (31.0)	674 (32.9)
Unknown	650 (2.6)	364 (2.3)	156 (2.8)	63 (2.9)	67 (3.3)
Any psychiatric disorder	2379 (9.4)	993 (6.4)	562 (10.2)	321 (14.8)	503 (24.6)
Alcohol or drug misuse disorder	595 (2.4)	234 (1.5)	120 (2.2)	87 (4.0)	154 (7.5)
Depressive disorder	1221 (4.8)	444 (2.9)	309 (5.6)	184 (8.5)	284 (13.9)
Anxiety disorder	1136 (4.5)	443 (2.9)	268 (4.8)	149 (6.9)	276 (13.5)
Stress-related disorder	744 (2.9)	275 (1.8)	169 (3.1)	106 (4.9)	194 (9.5)
Depressive symptoms, mean (SD)^a^	7.05 (5.64)	5.78 (4.82)	8.16 (5.72)	9.25 (6.17)	11.1 (7.03)

Abbreviations: ACE, adverse childhood experience; DZ, dizygotic; MZ, monozygotic.

^a^ Depressive symptoms were measured with the Center for Epidemiologic Studies Depression Scale. All items were answered on a 4-point scale and then summed to create a total score ranging from 0 to 33, with higher scores indicating greater levels of depressive symptoms.

A total of 2379 twins (9.4%) received a clinical diagnosis of any psychiatric disorder during the study period (Table 1). The incidence of any psychiatric disorder increased from 993 individuals (6.4%) among participants without any ACEs to 503 individuals (24.6%) among those reporting 3 or more ACEs (Table 1); the pattern was also present for each subcategory and for depressive symptoms.

At the cohort level, a greater number of ACEs was associated with increased odds of any psychiatric disorder in a dose-dependent manner (Table 2). Every additional ACE was associated with 52% greater odds of any psychiatric disorder (OR, 1.52; 95% CI, 1.48-1.57). The same pattern was noted among males and females (eTable 3 in Supplement 1). Discordant twin pair analyses revealed statistically significant but lower increases in odds of any psychiatric disorder associated with every additional ACE (DZ twin pairs: OR, 1.29; 95% CI, 1.14-1.47; MZ twin pairs: OR, 1.20; 95% CI, 1.02-1.40), corresponding to 39.1% and 57.5% attenuated ORs in DZ and MZ twins, respectively. Moreover, compared with 0 ACEs, exposure to 3 or more ACEs was associated with greater odds of any psychiatric disorder at the cohort level and in DZ and MZ twin pairs (Table 2). However, ORs in DZ and MZ twins were approximately half of the cohort-level OR (full cohort: 4.57; 95% CI, 4.05-5.15; DZ twin pairs: 2.25; 95% CI 1.41-3.61; MZ twin pairs: 2.11; 95% CI, 1.13-3.94). Similar results were observed for depressive symptoms (eTable 4 in Supplement 1) but with lower attenuation in estimates in discordant twin pair models.

**Table 2.** Associations Between No. of ACEs and Any Adult Psychiatric Disorder

Outcome	Model 1 (full cohort)^a^			Model 2 (in DZ twins)^a^				Model 3 (in MZ twins)^a^			
	Participants, No.		OR (95% CI)	Participants, No.			OR (95% CI)	Participants, No.			OR (95% CI)
	Total	With disorder^b^		Total^c^	With disorder^b^			Total^c^	With disorder^b^		
ACE total score^d^	25 252	2379	1.52 (1.48-1.57)	4018	402		1.29 (1.14-1.47)	2834	339		1.20 (1.02-1.40)
Any ACE, No.											
0	15 501	993	1 [Reference]	1554	100	1 [Reference]		1010	88	1 [Reference]	
≥1	9751	1386	2.39 (2.19-2.60)	1554	167	1.72 (1.31-2.28)		1010	99	1.19 (0.84-1.70)	
No. of ACEs											
0	15 501	993	1 [Reference]	1554	100	1 [Reference]		1010	88	1 [Reference]	
1	5531	562	1.65 (1.48-1.84)	1077	95	1.46 (1.07-1.98)		724	62	1.17 (0.80-1.72)	
2	2174	321	2.47 (2.16-2.83)	301	40	2.37 (1.52-3.70)		189	19	0.84 (0.50-1.42)	
≥3	2046	503	4.57 (4.05-5.15)	176	32	2.25 (1.41-3.61)		97	17	2.11 (1.13-3.94)	

Abbreviations: ACE, adverse childhood experience; DZ, dizygotic; MZ, monozygotic; OR, odds ratio.

^a^ Models were adjusted for age and sex; 95% CIs are based on robust SEs, calculated using generalized estimating equation analyses.

^b^ Any adult psychiatric disorder.

^c^ Total equals the number of individual twins from exposure-discordant twin pairs in the analysis.

^d^ ORs are for every additional ACE.

**Figure 2.**
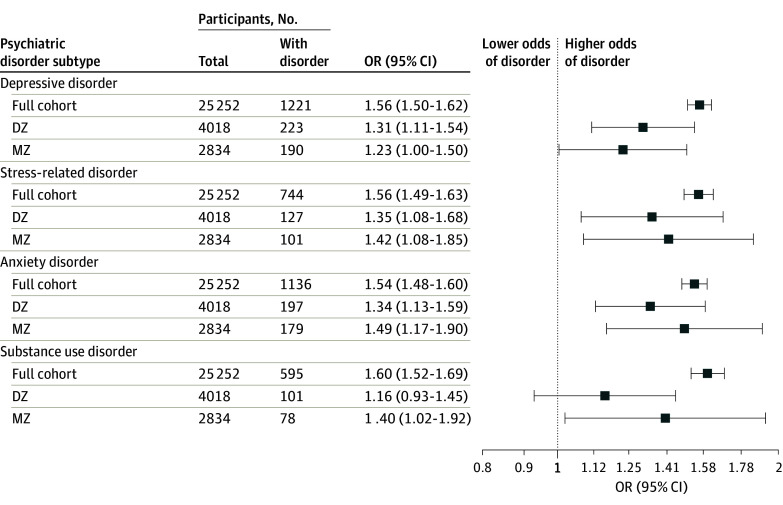
Associations Between Number of Adverse Childhood Experiences and Adult Psychiatric Disorder Subtypes Associations are given in the full cohort and in exposure-discordant twin pairs; odds ratios (ORs) are per additional ACE. Models were adjusted for age and sex; 95% CIs are based on robust standard errors calculated using generalized estimating equations. DZ indicates dizygotic twins; MZ, monozygotic twins; OR, odds ratio.

We observed associations between a greater number of ACEs and increased odds of depressive disorders, anxiety disorders, and stress-related disorders at the cohort level and in discordant twin pair models (Figure 2). However, there was an association with substance use disorder in MZ twin pairs but not in DZ twin pairs (Figure 2).

**Figure 3.**
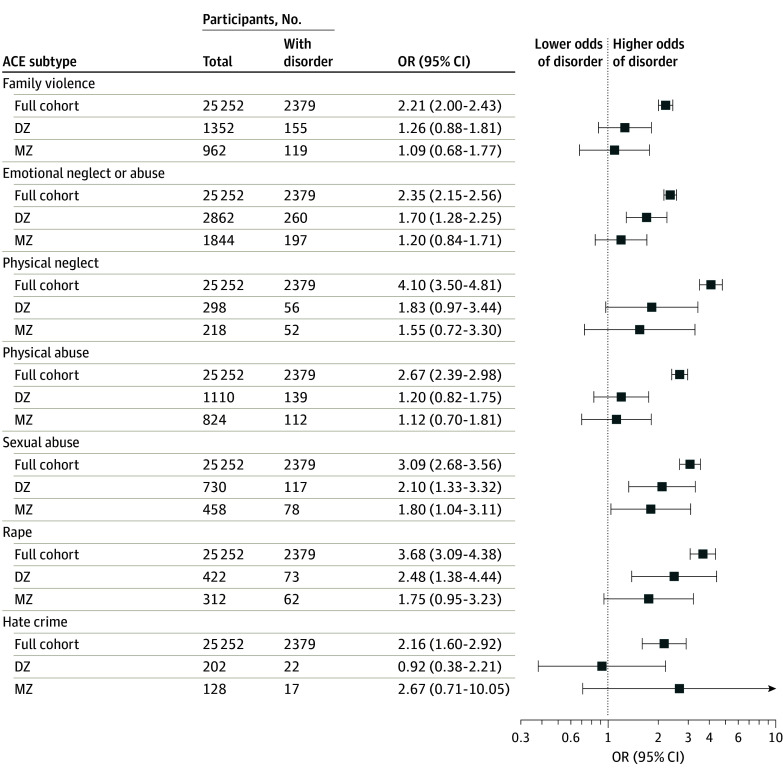
Associations Between Adverse Childhood Experience (ACE) Subtypes and Any Adult Psychiatric Disorder Associations are given in the full cohort and in exposure-discordant twin pairs; odds ratios (ORs) are for any ACE vs no ACE. Models were adjusted for age and sex; 95% CIs are based on robust standard errors calculated using generalized estimating equations. DZ indicates dizygotic twins; MZ, monozygotic twins.

All ACE subtypes were associated with increased odds of any psychiatric disorder in cohort-level analyses (Figure 3). Proportions of twin pair ACE discordance varied from 676 of 1080 twins with exposure (62.6%) on family violence to 211 of 227 twins with exposure (93.0%) on rape for DZ twin pairs and from 481 of 1045 twins with exposure (46.0%) on family violence to 156 of 180 twins with exposure (86.7%) on rape for MZ twin pairs (eTable 5 in Supplement 1). In discordant twin pair analyses, point estimates were considerably attenuated for all ACE subtypes except sexual abuse, for which there remained an association in all comparisons; ORs for individuals who were exposed to childhood sexual abuse compared with those who were not exposed were 3.09 (95% CI, 2.68-3.56) in the full cohort, 2.10 (95% CI, 1.33-3.32) in DZ twin pairs, and 1.80 (95% CI, 1.04-3.11) in MZ twin pairs (Figure 3). In contrast, the association between ACE subtypes and depressive symptoms remained in all discordant twin pair models except for physical abuse in MZ twin pairs (eFigure 3 in Supplement 1).

In sensitivity analyses, the pattern of associations remained the same when the youngest cohort (CATSS) was excluded from analyses (eTable 6 in Supplement 1), while estimates remained larger in the 2 youngest cohorts (YATSS and CATSS) (eTable 7 in Supplement 1) than in the oldest cohort (STAGE) (eTable 8 in Supplement 1). A similar pattern of associations was found when dispensed psychotropic medications were included as an indication for milder psychiatric disorders (eTable 9 in Supplement 1). Excluding participants with any psychiatric disorder prior to age 19 years revealed largely similar estimates, although with lower precision (eTable 10 in Supplement 1). Moreover, restricting follow-up of psychiatric disorder diagnosis to the date after the ACE assessment yielded a similar pattern of associations at the cohort level and in discordant DZ twin pair analysis, while estimates had low precision and were substantially diluted in the MZ twin pair analysis (eTable 1 in Supplement 1).

## Discussion

In this Swedish cohort study using twin data, we found that ACEs were associated with adult mental health outcomes after adjustment for familial confounding. That is, after adjusting for shared genetic and environmental factors in stringent twin analyses, the association between ACEs and clinically confirmed adult psychiatric disorders remained evident, with particularly large increases in odds after multiple ACEs or sexual abuse. However, attenuated estimates in DZ and MZ twin pairs suggest that familial confounding also contributed to the ACE-mental health association.

In line with a large body of research,^9,10,17,48^ we found that exposure to ACEs was phenotypically associated with adult mental health outcomes in a dose-dependent manner. Complementing previous work, we observed that exposure to ACEs was associated with increased odds of clinically confirmed adult psychiatric disorders and increased levels of self-reported depressive symptoms after adjusting for familial confounding due to shared genetic and environmental factors. However, the attenuation of effect sizes from the full cohort to DZ twins (39.1%) and MZ twins (57.5%) also suggests that familial confounding contributed to the association between ACEs and adult mental health outcomes. In other words, our results indicate that childhood environmental conditions (eg, parental education level and other socioeconomic conditions) and genetic predisposition may have contributed to the association between ACEs and adult mental health outcomes. However, further research is needed to determine which factors (genetic factors, early environmental factors, or both) are associated with increases in the risk of adult psychiatric disorders among individuals who experienced ACEs.

Our findings are broadly consistent with previous cross-sectional discordant twin pair studies, which reported that familial confounding contributed at least partly to associations between ACEs and self-reported alcohol dependence, personality disorders, and ADHD symptoms in adulthood.^30,31,32,33,36^ In addition, our results are in line with those of a 2023 meta-analysis^49^ reporting associations between ACEs and modest increases in risks of mental health problems (ie, assessed mostly in adolescence and young adulthood) after quasi-experimental adjustment for unmeasured confounders. Our study complements this literature with prospectively ascertained data on clinically confirmed adult psychiatric disorders, also yielding findings that associations between ACEs and psychiatric conditions in adulthood remained after adjustment for underlying shared confounders.

Although few estimates were significant between distinct types of ACEs and adult psychiatric disorders in the most stringent MZ analyses, our results demonstrated that familial factors contributed to a lesser extent to the association between sexual abuse and adult psychiatric disorders. This is in line with previous discordant twin pair research consistently indicating independent associations of child sexual abuse with self-assessed depression, anxiety, and substance abuse.^34,35,50,51^ Furthermore, we observed that MZ twins exposed to multiple (≥3) ACEs remained more likely compared with their co-twins who were not exposed to be diagnosed with a psychiatric disorder in adulthood. These results are consistent with increasing evidence on the accumulation model of ACEs.^9,52^

### Strengths and Limitations

The main strengths of this study include the large, nationwide twin sample and use of prospectively collected data on clinically confirmed psychiatric disorders, ascertained independently from ACE assessments. However, results of the study should be considered in the context of several limitations. First, although the discordant twin pair design inherently adjusts for shared environmental conditions, it is possible that some unmeasured factors that differed between twins, such as the rare occurrence of birth complications of 1 twin, contributed to observed estimates. Another important limitation is that ACEs were retrospectively reported and may thus be subject to recall bias.^53^ This is a common approach for assessing ACEs, but we cannot exclude the possibility that current mental health status influenced the reporting of ACEs.^54,55^ Although the agreement between retrospective and prospective measures of ACEs has been reported to be low,^56^ both have been associated with adverse adult health outcomes.^54^ Indeed, a 2023 meta-analysis^49^ found no differences in associations between retrospective and prospective ACE measures and mental health problems. In our study, estimates observed for associations between ACEs and current depressive symptoms may certainly have been affected by recall bias; however, estimates of clinically confirmed psychiatric disorders may be less vulnerable to such bias.^54^ This approach (ie, combining retrospectively reported ACEs and independently ascertained, clinically confirmed psychiatric disorders), as conducted in this study, has been recommended in previous literature.^54^

Similarly, given that our assessment of ACEs was based on selected items from the LSC-R deemed most relevant for the Swedish context, our findings are not directly comparable to findings of other studies from other countries and cultures with different sets of ACE items. Comparisons of ACE prevalence across cultures and populations is already complicated for several other reasons; for example, Sweden was the first country to legally ban physical punishment of children, in 1979.^57^ Furthermore, twin pair discordance on specific ACEs varied considerably, from 46.0% for family violence in MZ twins to 93.0% for rape in DZ twins; this may reflect the varying familial nature of these exposures. Although family violence had the lowest twin discordance of all ACEs, it may still seem counterintuitive that 46.0% of MZ twins were discordant on family violence given that this exposure unquestionably takes place in the family environment. It should be noted, however, that the question on family violence refers to an incident (until age 18 years) in which the respondent witnessed violence between family members. Although twin pairs are likely to have witnessed a chronic pattern of family violence (ie, repeated events), sporadic incidences of family violence may be witnessed by 1 but not the other twin.

In addition, given that we identified psychiatric disorders through inpatient or specialized outpatient hospital visits, we captured a small proportion of all psychiatric disorders (ie, only the most severe cases). This may have further led us to underestimate the proportion of twins with prevalent psychiatric disorders at age 19 years. Importantly, we were unable to include individuals seeking primary care only; however, this concern was to some extent alleviated given similar results of a sensitivity analyses in which we additionally considered the use of dispensed psychotropic medications as an indication of psychiatric disorders covered in primary care. Furthermore, it should be noted that while nonresponse may have led to some bias, previous attrition analyses of the Swedish Twin Registry cohorts indicated minor differences between responders and nonresponders.^40,58^ Additionally, although our study included a large sample of Swedish twins, it remains unknown whether the results can be generalized outside of Nordic countries.

## Conclusions

This cohort study using twin data found that the association between ACEs and adult mental health outcomes remained after adjusting for familial confounding due to shared genetic and environmental factors. This suggests that interventions targeting ACEs, including primary prevention and enhanced access to evidence-based trauma therapies to individuals who experienced ACEs, may be associated with reduced risk of future psychopathology. However, our findings additionally indicate that family-wide risk factors (eg, genetic predisposition and socioeconomic disadvantage in childhood) also contributed to adult mental health outcomes among individuals who experienced ACEs, suggesting that there may be added value in addressing risk factors within the whole family.

## Notes

Supplement 1. **eTable 1.** Items Applied From the Life Stressor Checklist-Revised to Assess Adverse Childhood Experiences in the Swedish Twin Registry **eTable 2.** International Classification of Diseases (ICD) Codes Used to Identify Psychiatric Disorders **eTable 3.** Associations Between No. of Adverse Childhood Experiences and Any Psychiatric Disorder in full Cohort by Sex **eTable 4.** Associations Between No. of Adverse Childhood Experiences and Symptoms of Depression in Full Cohort and in Exposure-Discordant Twin Pairs **eTable 5.** No. of Twin Individuals in Full Twin Pairs, Exposed Twin Individuals, and Exposure-Discordant Twins **eTable 6.** Associations Between No. of Adverse Childhood Experiences and Any Diagnosed Psychiatric Disorder in Full Cohort and in Exposure-Discordant Twin Pairs Excluding the Child and Adolescent Twin Study in Sweden **eTable 7.** Associations Between No. of Adverse Childhood Experiences and Any Diagnosed Psychiatric Disorder in Full Cohort and in Exposure-Discordant Twin Pairs Excluding the Study of Twin Adults: Genes and Environment **eTable 8.** Associations Between No. of Adverse Childhood Experiences and Any Diagnosed Psychiatric Disorder in Full Cohort and in Exposure-Discordant Twin Pairs in the Study of Twin Adults: Genes and Environment **eTable 9.** Associations Between No. of Adverse Childhood Experiences and Any Diagnosed Psychiatric Disorder Including Dispensed Psychotropic Medications as Indication for Mild Psychiatric Disorder Not Attended by Specialist Care in full Cohort and in Exposure-Discordant Twin Pairs **eTable 10.** Associations Between No. of Adverse Childhood Experiences and Any Diagnosed Psychiatric Disorder in Full Cohort and in Exposure-Discordant Twin Pairs Excluding Twins Diagnosed With Any Psychiatric Disorder Before Age 19 y **eTable 11.** Associations Between No. of Adverse Childhood Experiences (ACEs) and Any Diagnosed Psychiatric Disorder in Full Cohort and in Exposure-Discordant Twin Pairs Excluding Twins Diagnosed With Any Psychiatric Disorder Before Answering web-Based Survey With ACE Assessment **eFigure 1.** Flowchart of Analytic Sample Excluding Twins Diagnosed With Any Psychiatric Disorder Before Age 19 y **eFigure 2.** Flowchart of Analytic Sample Where Follow-Up for Psychiatric Disorders Starts After Web-Based Surveys With Adverse Childhood Experience Assessment Excluding Twins Who Received Diagnosis of Any Psychiatric Disorder Before Answering Survey **eFigure 3.** Associations Between Adverse Childhood Experience Subtypes and Depression Symptoms in Full Cohort and Within Exposure-Discordant Twin Pairs **eTable 1.** Items Applied From Life Stressor Checklist-Revised Plus Hate Crime to Assess Adverse Childhood Experiences in the Swedish Twin Registry

Supplement 2.
Data Sharing Statement

## References

[1] Stoltenborgh M, Bakermans-Kranenburg MJ, Alink LRA, van Ijzendoorn MH. The prevalence of child maltreatment across the globe: review of a series of meta-analyses. Child Abuse Rev. 2015;24:37-50. doi:10.1002/car.2353

[2] Moody G, Cannings-John R, Hood K, Kemp A, Robling M. Establishing the international prevalence of self-reported child maltreatment: a systematic review by maltreatment type and gender. BMC Public Health. 2018;18(1):1164. doi:10.1186/s12889-018-6044-y 30305071 PMC6180456

[3] Merrick MT, Ford DC, Ports KA, Guinn AS. Prevalence of adverse childhood experiences from the 2011-2014 Behavioral Risk Factor Surveillance System in 23 states. JAMA Pediatr. 2018;172(11):1038-1044. doi:10.1001/jamapediatrics.2018.2537 30242348 PMC6248156

[4] Gilbert R, Widom CS, Browne K, Fergusson D, Webb E, Janson S. Burden and consequences of child maltreatment in high-income countries. Lancet. 2009;373(9657):68-81. doi:10.1016/S0140-6736(08)61706-7 19056114

[5] Degli Esposti M, Humphreys DK, Jenkins BM, . Long-term trends in child maltreatment in England and Wales, 1858-2016: an observational, time-series analysis. Lancet Public Health. 2019;4(3):e148-e158. doi:10.1016/S2468-2667(19)30002-7 30851868

[6] Anda RF, Butchart A, Felitti VJ, Brown DW. Building a framework for global surveillance of the public health implications of adverse childhood experiences. Am J Prev Med. 2010;39(1):93-98. doi:10.1016/j.amepre.2010.03.015 20547282

[7] Anda RF, Felitti VJ, Bremner JD, . The enduring effects of abuse and related adverse experiences in childhood: a convergence of evidence from neurobiology and epidemiology. Eur Arch Psychiatry Clin Neurosci. 2006;256(3):174-186. doi:10.1007/s00406-005-0624-4 16311898 PMC3232061

[8] Bellis MA, Hughes K, Leckenby N, Hardcastle KA, Perkins C, Lowey H. Measuring mortality and the burden of adult disease associated with adverse childhood experiences in England: a national survey. J Public Health (Oxf). 2015;37(3):445-454. doi:10.1093/pubmed/fdu065 25174044 PMC4552010

[9] Hughes K, Bellis MA, Hardcastle KA, . The effect of multiple adverse childhood experiences on health: a systematic review and meta-analysis. Lancet Public Health. 2017;2(8):e356-e366. doi:10.1016/S2468-2667(17)30118-4 29253477

[10] Petruccelli K, Davis J, Berman T. Adverse childhood experiences and associated health outcomes: a systematic review and meta-analysis. Child Abuse Negl. 2019;97:104127. doi:10.1016/j.chiabu.2019.104127 31454589

[11] Scott KM, Smith DR, Ellis PM. Prospectively ascertained child maltreatment and its association with *DSM-IV* mental disorders in young adults. Arch Gen Psychiatry. 2010;67(7):712-719. doi:10.1001/archgenpsychiatry.2010.71 20603452

[12] McLaughlin KA, Koenen KC, Bromet EJ, . Childhood adversities and post-traumatic stress disorder: evidence for stress sensitisation in the World Mental Health Surveys. Br J Psychiatry. 2017;211(5):280-288. doi:10.1192/bjp.bp.116.197640 28935660 PMC5663970

[13] Nelson J, Klumparendt A, Doebler P, Ehring T. Childhood maltreatment and characteristics of adult depression: meta-analysis. Br J Psychiatry. 2017;210(2):96-104. doi:10.1192/bjp.bp.115.180752 27908895

[14] Chapman DP, Whitfield CL, Felitti VJ, Dube SR, Edwards VJ, Anda RF. Adverse childhood experiences and the risk of depressive disorders in adulthood. J Affect Disord. 2004;82(2):217-225. doi:10.1016/j.jad.2003.12.013 15488250

[15] Mandelli L, Petrelli C, Serretti A. The role of specific early trauma in adult depression: a meta-analysis of published literature: childhood trauma and adult depression. Eur Psychiatry. 2015;30(6):665-680. doi:10.1016/j.eurpsy.2015.04.007 26078093

[16] Li M, D’Arcy C, Meng X. Maltreatment in childhood substantially increases the risk of adult depression and anxiety in prospective cohort studies: systematic review, meta-analysis, and proportional attributable fractions. Psychol Med. 2016;46(4):717-730. doi:10.1017/S0033291715002743 26708271

[17] Bellis MA, Hughes K, Ford K, Ramos Rodriguez G, Sethi D, Passmore J. Life course health consequences and associated annual costs of adverse childhood experiences across Europe and North America: a systematic review and meta-analysis. Lancet Public Health. 2019;4(10):e517-e528. doi:10.1016/S2468-2667(19)30145-8 31492648 PMC7098477

[18] Leza L, Siria S, López-Goñi JJ, Fernández-Montalvo J. Adverse childhood experiences (ACEs) and substance use disorder (SUD): a scoping review. Drug Alcohol Depend. 2021;221:108563. doi:10.1016/j.drugalcdep.2021.108563 33561668

[19] Narayan AJ, Lieberman AF, Masten AS. Intergenerational transmission and prevention of adverse childhood experiences (ACEs). Clin Psychol Rev. 2021;85:101997. doi:10.1016/j.cpr.2021.101997 33689982

[20] Moog NK, Cummings PD, Jackson KL, ; ECHO collaborators. Intergenerational transmission of the effects of maternal exposure to childhood maltreatment in the USA: a retrospective cohort study. Lancet Public Health. 2023;8(3):e226-e237. doi:10.1016/S2468-2667(23)00025-7 36841563 PMC9982823

[21] Sidebotham P, Golding J; ALSPAC Study Team. Avon Longitudinal Study of Parents and Children. Child maltreatment in the “children of the nineties” a longitudinal study of parental risk factors. Child Abuse Negl. 2001;25(9):1177-1200. doi:10.1016/S0145-2134(01)00261-7 11700691

[22] Polderman TJ, Benyamin B, de Leeuw CA, . Meta-analysis of the heritability of human traits based on fifty years of twin studies. Nat Genet. 2015;47(7):702-709. doi:10.1038/ng.3285 25985137

[23] Pezzoli P, Antfolk J, Hatoum AS, Santtila P. Genetic vulnerability to experiencing child maltreatment. Front Genet. 2019;10:852. doi:10.3389/fgene.2019.00852 31608106 PMC6758596

[24] Fisher HL, Caspi A, Moffitt TE, . Measuring adolescents’ exposure to victimization: The Environmental Risk (E-Risk) Longitudinal Twin Study. Dev Psychopathol. 2015;27(4 Pt 2):1399-1416. doi:10.1017/S0954579415000838 26535933 PMC4778729

[25] Pittner K, Bakermans-Kranenburg MJ, Alink LRA, . Estimating the heritability of experiencing child maltreatment in an extended family design. Child Maltreat. 2020;25(3):289-299. doi:10.1177/1077559519888587 31773993 PMC7370654

[26] Baldwin JR, Sallis HM, Schoeler T, . A genetically informed registered report on adverse childhood experiences and mental health. Nat Hum Behav. 2023;7(2):269-290. doi:10.1038/s41562-022-01482-9 36482079 PMC7614239

[27] Wang D, Jiang Q, Yang Z, Choi JK. The longitudinal influences of adverse childhood experiences and positive childhood experiences at family, school, and neighborhood on adolescent depression and anxiety. J Affect Disord. 2021;292:542-551. doi:10.1016/j.jad.2021.05.108 34147966

[28] Vitaro F, Brendgen M, Arseneault L. The discordant MZ-twin method: one step closer to the holy grail of causality. Int J Behav Dev. 2009;33(4):376-382. doi:10.1177/0165025409340805

[29] McGue M, Osler M, Christensen K. Causal inference and observational research: the utility of twins. Perspect Psychol Sci. 2010;5(5):546-556. doi:10.1177/1745691610383511 21593989 PMC3094752

[30] Magnusson Å, Lundholm C, Göransson M, Copeland W, Heilig M, Pedersen NL. Familial influence and childhood trauma in female alcoholism. Psychol Med. 2012;42(2):381-389. doi:10.1017/S0033291711001310 21798111 PMC3648622

[31] Young-Wolff KC, Kendler KS, Ericson ML, Prescott CA. Accounting for the association between childhood maltreatment and alcohol-use disorders in males: a twin study. Psychol Med. 2011;41(1):59-70. doi:10.1017/S0033291710000425 20346194 PMC3010204

[32] Capusan AJ, Kuja-Halkola R, Bendtsen P, . Childhood maltreatment and attention deficit hyperactivity disorder symptoms in adults: a large twin study. Psychol Med. 2016;46(12):2637-2646. doi:10.1017/S0033291716001021 27376862

[33] Alemany S, Goldberg X, van Winkel R, Gastó C, Peralta V, Fañanás L. Childhood adversity and psychosis: examining whether the association is due to genetic confounding using a monozygotic twin differences approach. Eur Psychiatry. 2013;28(4):207-212. doi:10.1016/j.eurpsy.2012.03.001 22944339

[34] Kendler KS, Bulik CM, Silberg J, Hettema JM, Myers J, Prescott CA. Childhood sexual abuse and adult psychiatric and substance use disorders in women: an epidemiological and cotwin control analysis. Arch Gen Psychiatry. 2000;57(10):953-959. doi:10.1001/archpsyc.57.10.953 11015813

[35] Nelson EC, Heath AC, Madden PA, . Association between self-reported childhood sexual abuse and adverse psychosocial outcomes: results from a twin study. Arch Gen Psychiatry. 2002;59(2):139-145. doi:10.1001/archpsyc.59.2.139 11825135

[36] Bornovalova MA, Huibregtse BM, Hicks BM, Keyes M, McGue M, Iacono W. Tests of a direct effect of childhood abuse on adult borderline personality disorder traits: a longitudinal discordant twin design. J Abnorm Psychol. 2013;122(1):180-194. doi:10.1037/a0028328 22686871 PMC3482426

[37] Furberg H, Lichtenstein P, Pedersen NL, . The STAGE cohort: a prospective study of tobacco use among Swedish twins. Nicotine Tob Res. 2008;10(12):1727-1735. doi:10.1080/14622200802443551 18988069 PMC2914543

[38] Lichtenstein P, Sullivan PF, Cnattingius S, . The Swedish Twin Registry in the third millennium: an update. Twin Res Hum Genet. 2006;9(6):875-882. doi:10.1375/twin.9.6.875 17254424

[39] Zagai U, Lichtenstein P, Pedersen NL, Magnusson PKE. The Swedish Twin Registry: content and management as a research infrastructure. Twin Res Hum Genet. 2019;22(6):672-680. doi:10.1017/thg.2019.99 31747977

[40] Anckarsäter H, Lundström S, Kollberg L, . The child and adolescent twin study in Sweden (CATSS). Twin Res Hum Genet. 2011;14(6):495-508. doi:10.1375/twin.14.6.495 22506305

[41] Wolfe J, Kimerling R. Gender issues in the assessment of posttraumatic stress disorder. In: Wilson JP, Keane TM, eds. Assessing Psychological Trauma and PTSD. The Guilford Press; 1997:192-238. Accessed January 31, 2024. https://psycnet.apa.org/record/1997-97162-007

[42] McHugo GJ, Caspi Y, Kammerer N, . The assessment of trauma history in women with co-occurring substance abuse and mental disorders and a history of interpersonal violence. J Behav Health Serv Res. 2005;32(2):113-127. doi:10.1007/BF02287261 15834262

[43] Ludvigsson JF, Andersson E, Ekbom A, . External review and validation of the Swedish National Inpatient Register. BMC Public Health. 2011;11:450. doi:10.1186/1471-2458-11-450 21658213 PMC3142234

[44] Carpenter JS, Andrykowski MA, Wilson J, . Psychometrics for two short forms of the Center for Epidemiologic Studies-Depression Scale. Issues Ment Health Nurs. 1998;19(5):481-494. doi:10.1080/016128498248917 9782864

[45] Neale MC, Hunter MD, Pritikin JN, . OpenMx 2.0: extended structural equation and statistical modeling. Psychometrika. 2016;81(2):535-549. doi:10.1007/s11336-014-9435-8 25622929 PMC4516707

[46] Zetterqvist J, Sjölander A. Doubly robust estimation with the R package drgee. Epidemiol Methods. 2015;4(1):69-86. doi:10.1515/em-2014-0021

[47] Wettermark B, Hammar N, Fored CM, . The new Swedish Prescribed Drug Register—opportunities for pharmacoepidemiological research and experience from the first six months. Pharmacoepidemiol Drug Saf. 2007;16(7):726-735. doi:10.1002/pds.1294 16897791

[48] Copeland WE, Shanahan L, Hinesley J, . Association of childhood trauma exposure with adult psychiatric disorders and functional outcomes. JAMA Netw Open. 2018;1(7):e184493. doi:10.1001/jamanetworkopen.2018.4493 30646356 PMC6324370

[49] Baldwin JR, Wang B, Karwatowska L, . Childhood maltreatment and mental health problems: a systematic review and meta-analysis of quasi-experimental studies. Am J Psychiatry. 2023;180(2):117-126. doi:10.1176/appi.ajp.20220174 36628513 PMC7614155

[50] Nelson EC, Heath AC, Lynskey MT, . Childhood sexual abuse and risks for licit and illicit drug-related outcomes: a twin study. Psychol Med. 2006;36(10):1473-1483. doi:10.1017/S0033291706008397 16854248

[51] Dinwiddie S, Heath AC, Dunne MP, . Early sexual abuse and lifetime psychopathology: a co-twin-control study. Psychol Med. 2000;30(1):41-52. doi:10.1017/S0033291799001373 10722174

[52] Dunn EC, Soare TW, Raffeld MR, . What life course theoretical models best explain the relationship between exposure to childhood adversity and psychopathology symptoms: recency, accumulation, or sensitive periods? Psychol Med. 2018;48(15):2562-2572. doi:10.1017/S0033291718000181 29478418 PMC6109629

[53] Hardt J, Rutter M. Validity of adult retrospective reports of adverse childhood experiences: review of the evidence. J Child Psychol Psychiatry. 2004;45(2):260-273. doi:10.1111/j.1469-7610.2004.00218.x 14982240

[54] Reuben A, Moffitt TE, Caspi A, . Lest we forget: comparing retrospective and prospective assessments of adverse childhood experiences in the prediction of adult health. J Child Psychol Psychiatry. 2016;57(10):1103-1112. doi:10.1111/jcpp.12621 27647050 PMC5234278

[55] Danese A, Widom CS. Objective and subjective experiences of child maltreatment and their relationships with psychopathology. Nat Hum Behav. 2020;4(8):811-818. doi:10.1038/s41562-020-0880-3 32424258

[56] Baldwin JR, Reuben A, Newbury JB, Danese A. Agreement between prospective and retrospective measures of childhood maltreatment: a systematic review and meta-analysis. JAMA Psychiatry. 2019;76(6):584-593. doi:10.1001/jamapsychiatry.2019.0097 30892562 PMC6551848

[57] Durrant JE. Evaluating the success of Sweden’s corporal punishment ban. Child Abuse Negl. 1999;23(5):435-448. doi:10.1016/S0145-2134(99)00021-6 10348380

[58] Pettersson E, Larsson H, D’Onofrio BM, Lichtenstein P. Associations between general and specific psychopathology factors and 10-year clinically relevant outcomes in adult Swedish twins and siblings. JAMA Psychiatry. 2023;80(7):728-737. doi:10.1001/jamapsychiatry.2023.1162 37163290 PMC10173102

