# Supplementary material for: Adverse Childhood Experiences and Adult Mental Health Outcomes
Source: JAMA Psychiatry. 2024 Mar 6;81(6):586–94. doi: 10.1001/jamapsychiatry.2024.0039 (PMC10918580; doi:10.1001/jamapsychiatry.2024.0039)
Supplement: Supplement 1. — eTable 1. Items Applied From the Life Stressor Checklist-Revised to Assess Adverse Childhood Experiences in the Swedish Twin Registry eTable 2. International Classification of Diseases (ICD) Codes Used to Identify Psychiatric Disorders eTable 3. Associations Between No. of Adverse Childhood Experiences and Any Psychiatric Disorder in full Cohort by Sex eTable 4. Associations Between No. of Adverse Childhood Experiences and Symptoms of Depression in Full Cohort and in Exposure-Discordant Twin Pairs eTable 5. No. of Twin Individuals in Full Twin Pairs, Exposed Twin Individuals, and Exposure-Discordant Twins eTable 6. Associations Between No. of Adverse Childhood Experiences and Any Diagnosed Psychiatric Disorder in Full Cohort and in Exposure-Discordant Twin Pairs Excluding the Child and Adolescent Twin Study in Sweden eTable 7. Associations Between No. of Adverse Childhood Experiences and Any Diagnosed Psychiatric Disorder in Full Cohort and in Exposure-Discordant Twin Pairs Excluding the Study of Twin Adults: Genes and Environment eTable 8. Associations Between No. of Adverse Childhood Experiences and Any Diagnosed Psychiatric Disorder in Full Cohort and in Exposure-Discordant Twin Pairs in the Study of Twin Adults: Genes and Environment eTable 9. Associations Between No. of Adverse Childhood Experiences and Any Diagnosed Psychiatric Disorder Including Dispensed Psychotropic Medications as Indication for Mild Psychiatric Disorder Not Attended by Specialist Care in full Cohort and in Exposure-Discordant Twin Pairs eTable 10. Associations Between No. of Adverse Childhood Experiences and Any Diagnosed Psychiatric Disorder in Full Cohort and in Exposure-Discordant Twin Pairs Excluding Twins Diagnosed With Any Psychiatric Disorder Before Age 19 y eTable 11. Associations Between No. of Adverse Childhood Experiences (ACEs) and Any Diagnosed Psychiatric Disorder in Full Cohort and in Exposure-Discordant Twin Pairs Excluding Twins Diagnosed With Any Psychiatric Disorder Befo [file jamapsychiatry-e240039-s001.pdf]

Supplemental Online Content

Daníelsdóttir HB, Aspelund T, Shen Q, et al. Adverse childhood experiences and adult mental health outcomes. *JAMA Psychiatry*. Published online March 6, 2024. doi:10.1001/jamapsychiatry.2024.0039

eTable 1. Items Applied From the Life Stressor Checklist-Revised to Assess Adverse Childhood Experiences in the Swedish Twin Registry

eTable 2. International Classification of Diseases (ICD) Codes Used to Identify Psychiatric Disorders

eTable 3. Associations Between No. of Adverse Childhood Experiences and Any Psychiatric Disorder in full Cohort by Sex

eTable 4. Associations Between No. of Adverse Childhood Experiences and Symptoms of Depression in Full Cohort and in Exposure-Discordant Twin Pairs

eTable 5. No. of Twin Individuals in Full Twin Pairs, Exposed Twin Individuals, and Exposure-Discordant Twins

eTable 6. Associations Between No. of Adverse Childhood Experiences and Any Diagnosed Psychiatric Disorder in Full Cohort and in Exposure-Discordant Twin Pairs Excluding the Child and Adolescent Twin Study in Sweden

eTable 7. Associations Between No. of Adverse Childhood Experiences and Any Diagnosed Psychiatric Disorder in Full Cohort and in Exposure-Discordant Twin Pairs Excluding the Study of Twin Adults: Genes and Environment

eTable 8. Associations Between No. of Adverse Childhood Experiences and Any Diagnosed Psychiatric Disorder in Full Cohort and in Exposure-Discordant Twin Pairs in the Study of Twin Adults: Genes and Environment

eTable 9. Associations Between No. of Adverse Childhood Experiences and Any Diagnosed Psychiatric Disorder Including Dispensed Psychotropic Medications as Indication for Mild Psychiatric Disorder Not Attended by Specialist Care in full Cohort and in Exposure-Discordant Twin Pairs

eTable 10. Associations Between No. of Adverse Childhood Experiences and Any Diagnosed Psychiatric Disorder in Full Cohort and in Exposure-Discordant Twin Pairs Excluding Twins Diagnosed With Any Psychiatric Disorder Before Age 19 y

© 2024 Daníelsdóttir HB et al. *JAMA Psychiatry.*

eTable 11. Associations Between No. of Adverse Childhood Experiences (ACEs) and Any Diagnosed Psychiatric Disorder in Full Cohort and in Exposure-Discordant Twin Pairs Excluding Twins Diagnosed With Any Psychiatric Disorder Before Answering web-Based Survey With ACE Assessment

eFigure 1. Flowchart of Analytic Sample Excluding Twins Diagnosed With Any Psychiatric Disorder Before Age 19 y

eFigure 2. Flowchart of Analytic Sample Where Follow-Up for Psychiatric Disorders Starts After Web-Based Surveys With Adverse Childhood Experience Assessment Excluding Twins Who Received Diagnosis of Any Psychiatric Disorder Before Answering Survey

eFigure 3. Associations Between Adverse Childhood Experience Subtypes and Depression Symptoms in Full Cohort and Within Exposure-Discordant Twin Pairs

eTable 1. Items Applied From Life Stressor Checklist-Revised Plus Hate Crime to Assess Adverse Childhood Experiences in the Swedish Twin Registry

This supplemental material has been provided by the authors to give readers additional information about their work.

© 2024 Daníelsdóttir HB et al. *JAMA Psychiatry.*

Type of ACE	Item
Family violence	When you were young, before age 18, did you ever see physical violence between family members? For example, hitting, kicking or punching?
Emotional neglect/abuse	Have you ever been emotionally abused or neglected – for example, being frequently shamed, embarrassed, ignored, or repeatedly told that you were “no good”?
Physical neglect	Have you ever been physically neglected – for example, not fed, not properly clothed, or left to take care of yourself when you felt you were too young or ill?
Physical abuse	Have you ever been physically abused – for example, hit, choked, burned, or beaten, or severely punished, for example, locked up in a closet, tied up, or chained – by someone you knew well such as a parent, sibling, boyfriend or girlfriend?
Sexual abuse	Were you ever touched or made to touch someone else in a sexual way, because you felt forced in some way or threatened by harm to yourself or someone else?
Rape	Did you ever have sex because you felt forced in some way or threatened by harm to yourself or someone else? With sex, we mean orally, anally, and/or genitally
Hate crime	Have you ever been victim of a hate crime? This could include being subject to violence because of your race, ethnicity, gender, sexual orientation or religion.

*Hate crime was not included in the original Life Stressor Checklist-revised.

© 2024 Daníelsdóttir HB et al. *JAMA Psychiatry.*

eTable 2. International Classification of Diseases (ICD) codes, eight (ICD-8; 1969-1986), ninth (ICD-9; 1987-1996), and tenth (ICD-10; 1997-2016) used to identify psychiatric disorders.

Psychiatric diagnosis	ICD-8	ICD-9	ICD-10
Alcohol or drug misuse disorders	303, 304	291E, 292.0, 303, 304, 305	F10-F19
Depressive disorders	296.0, 300.4	296, 311	F32-F34, F38-F39
Anxiety disorders	300.0, 300.1	300A, 300C, 300D	F40, F41, F42, F44
Stress-related disorders	307	308, 309	F43

Note. Based on the Swedish version of the ICD version 8, 9 and 10 codes for the identification of diagnosis.

© 2024 Daníelsdóttir HB et al. *JAMA Psychiatry.*

eTable 3. Associations between the number of ACEs and any psychiatric disorder in the full cohort, stratified by sex.

	Women			Men			*P* for interaction
	N	cases[a]	OR (95% CI)	N	cases	OR (95% CI)	
**ACE total score[b]**	15,038	1621	1.53 (1.48, 1.59)	10,214	758	1.49 (1.41, 1.59)	0.51
**Any ACE**							
0 ACE	8914	626	1.00 (ref.)	6587	367	1.00 (ref.)	0.39
≥ 1 ACE	6124	995	2.58 (2.32, 2.87)	3627	391	2.04 (1.76, 2.37)	
**Number of ACEs**							
0 ACE	8914	626	1.00 (ref.)	6587	365	1.00 (ref.)	0.37
1 ACE	3319	376	1.71 (1.49, 1.96)	2212	184	1.55 (1.29, 1.87)	
2 ACE	1385	240	2.79 (2.37, 3.28)	789	80	1.89 (1.46, 2.43)	
≥ 3 ACEs	1420	377	4.79 (4.14, 5.53)	626	126	4.14 (3.31, 5.18)	

ACEs = adverse childhood experiences; OR = Odds Ratio; CI = Confidence Interval.
Models were adjusted for age;
95% CIs are based on robust SEs, calculated using generalized estimating equations;
[a]Any adult psychiatric disorder
[b]OR for every additional ACE.

© 2024 Daníelsdóttir HB et al. *JAMA Psychiatry.*

eTable 4. Associations between the number of ACEs and symptoms of depression in the full cohort and within exposure discordant twin-pairs

	Model 1 (Full cohort)			Model 2 (Within DZ twins)			Model 3 (Within MZ twins)		
	N total	Mean	exp(β) (95% CI)	N[a]	Mean	exp(β) (95% CI)	N[a]	Mean	exp(β) (95% CI)
**ACE total score**[b]	28,941	7.05	1.21 (1.20, 1.22)	4980	8.13	1.18 (1.16, 1.21)	3184	7.87	1.12 (1.09, 1.15)
**Any ACE**									
0 ACE	17,569	5.78	1.00 (ref.)	1910	6.16	1.00 (ref.)	1123	6.35	1.00 (ref.)
≥ 1 ACE	11,372	9.02	1.52 (1.49, 1.55)	1910	8.80	1.39 (1.33, 1.45)	1123	7.82	1.22 (1.15, 1.28)
**Number of ACEs**									
0 ACE	17,569	5.78	1.00 (ref.)	1910	6.16	1.00 (ref.)	1123	6.35	1.00 (ref.)
1 ACE	6479	8.16	1.39 (1.36, 1.42)	1325	8.28	1.33 (1.27, 1.40)	804	7.38	1.18 (1.12, 1.25)
2 ACE	2520	9.25	1.57 (1.52, 1.62)	368	9.31	1.47 (1.36, 1.59)	212	8.25	1.27 (1.17, 1.39)
≥ 3 ACEs	2373	11.13	1.87 (1.81, 1.93)	217	11.14	1.65 (1.52, 1.80)	107	10.23	1.38 (1.25, 1.53)

ACEs = adverse childhood experiences; DZ = Dizygotic twins; MZ = Monozygotic twins; CI = Confidence Interval.
Models were adjusted for age and sex;
Estimates are given as the percentage increase in depression symptoms, for every one-unit increase in ACEs;
95% CIs are based on robust SEs, calculated using generalized estimating equations;
[a]Exposure discordant twins, the number of individual twins from exposure discordant twin pairs in the analysis;
[b]exp(β) for every additional ACE.

© 2024 Daníelsdóttir HB et al. *JAMA Psychiatry.*

eTable 5. Number of twin individuals in full twin-pairs, number of exposed twin individuals and number of exposure discordant twins.

	Family violence	Emotional neglect	Physical neglect	Physical abuse	Sexual abuse	Rape	Hate crime
N total MZ in full pairs	6876	6876	6876	6876	6876	6876	6876
N exposed MZ	1045	1822	183	624	303	180	82
N discordant MZ	481 (46%)	922 (51%)	109 (60%)	412 (66%)	229 (76%)	156 (87%)	64 (78%)
N total DZ in full pairs	8464	8464	8464	8464	8464	8464	8464
N exposed DZ	1080	2453	225	735	417	227	109
N discordant DZ	676 (63%)	1431 (58%)	149 (66%)	555 (75%)	365 (88%)	211 (93%)	101 (93%)

© 2024 Daníelsdóttir HB et al. *JAMA Psychiatry.*

eTable 6. Associations between the number of ACEs and any diagnosed psychiatric disorder in the full cohort and within exposure discordant twin-pairs in the STAGE and YATSS cohorts (excluding CATSS).

	Model 1 (Full cohort)			Model 2 (Within DZ twins)			Model 3 (Within MZ twins)		
	N total	cases[a]	OR (95% CI)	N[b]	cases	OR (95% CI)	N[b]	cases	OR (95% CI)
**ACE total score**[c]	21,465	2240	1.52 (1.47, 1.57)	3072	365	1.26 (1.10, 1.44)	2442	313	1.21 (1.02, 1.41)
**Any ACE**									
0 ACE	13,260	949	1.00 (ref.)	1181	96	1.00 (ref.)	861	83	1.00 (ref.)
≥ 1 ACE	8205	1291	2.39 (2.18, 2.61)	1181	153	1.65 (1.24, 2.20)	861	93	1.19 (0.83, 1.70)
**Number of ACEs**									
0 ACE	13,260	949	1.00 (ref.)	1181	96	1.00 (ref.)	861	83	1.00 (ref.)
1 ACE	4580	529	1.69 (1.51 1.87)	806	89	1.42 (1.03, 1.95)	617	60	1.14 (0.77, 1.69)
2 ACE	1833	298	2.47 (2.15, 2.85)	230	36	2.42 (1.51, 3.88)	159	18	0.88 (0.51, 1.51)
≥ 3 ACEs	1792	464	4.41 (3.90, 5.00)	145	28	1.90 (1.16, 3.11)	85	15	2.07 (1.10, 3.87)

ACEs = adverse childhood experiences; DZ = Dizygotic twins; MZ = Monozygotic twins; OR = Odds Ratio; CI = Confidence Interval.
Models were adjusted for age and sex;
95% CIs are based on robust SEs, calculated using generalized estimating equations;
[a]Any adult psychiatric disorder
[b]Exposure discordant twins, the number of individual twins from exposure discordant twin pairs in the analysis;
[c]OR for every additional ACE.

© 2024 Daníelsdóttir HB et al. *JAMA Psychiatry.*

eTable 7. Associations between the number of ACEs and any adult psychiatric disorder in the full cohort and within exposure discordant twin-pairs in the YATSS and CATSS cohorts (excluding STAGE).

	Model 1 (Full cohort)			Model 2 (Within DZ twins)			Model 3 (Within MZ twins)		
	N total	cases[a]	OR (95% CI)	N[b]	cases	OR (95% CI)	N[b]	cases	OR (95% CI)
**ACE total score[c]**	7996	540	1.62 (1.52, 1.72)	1436	106	1.48 (1.14, 1.93)	868	73	1.58 (0.99, 2.51)
**Any ACE**									
0 ACE	5125	220	1.00 (ref.)	573	22	1.00 (ref.)	321	13	1.00 (ref.)
≥ 1 ACE	2871	320	3.04 (2.52, 2.66)	573	47	2.30 (1.31, 4.03)	321	23	2.43 (1.01, 5.86)
**Number of ACEs**									
0 ACE	5125	220	1.00 (ref.)	573	22	1.00 (ref.)	321	13	1.00 (ref.)
1 ACE	1652	115	1.88 (1.47 2.40)	403	22	1.53 (0.81, 2.90)	219	13	2.44 (0.92, 6.46)
2 ACE	634	74	3.17 (2.39, 4.20)	110	13	3.85 (1.51, 9.84)	61	5	1.15 (0.32, 4.08)
≥ 3 ACEs	585	131	6.34 (4.96, 8.09)	60	12	4.42 (1.74, 11.24)	41	5	7.35 (1.04, 52.18)

ACEs = adverse childhood experiences; DZ = Dizygotic twins; MZ = Monozygotic twins; OR = Odds Ratio; CI = Confidence Interval.
Models were adjusted for age and sex;
95% CIs are based on robust SEs, calculated using generalized estimating equations;
[a]Any adult psychiatric disorder
[b]Exposure discordant twins, the number of individual twins from exposure discordant twin pairs in the analysis;
[c]OR for every additional ACE.

© 2024 Daníelsdóttir HB et al. *JAMA Psychiatry.*

eTable 8. Associations between the number of ACEs and any adult psychiatric disorder in the full cohort and within exposure discordant twin-pairs in the STAGE cohort.

	Model 1 (Full cohort)			Model 2 (Within DZ twins)			Model 3 (Within MZ twins)		
	N total	cases[a]	OR (95% CI)	N[b]	cases	OR (95% CI)	N[b]	cases	OR (95% CI)
**ACE total score**[c]	17,256	1839	1.50 (1.44, 1.55)	2582	296	1.22 (1.05, 1.42)	1966	266	1.12 (0.95, 1.34)
**Any ACE**									
0 ACE	10,376	773	1.00 (ref.)	981	78	1.00 (ref.)	689	75	1.00 (ref.)
≥ 1 ACE	6880	1066	2.25 (2.04, 2.48)	981	120	1.56 (1.13, 2.17)	689	76	1.02 (0.69, 1.51)
**Number of ACEs**									
0 ACE	10,376	773	1.00 (ref.)	981	78	1.00 (ref.)	689	75	1.00 (ref.)
1 ACE	3879	447	1.61 (1.42, 1.82)	674	73	1.43 (1.00, 2.03)	505	50	1.01 (0.67, 1.54)
2 ACE	1540	247	2.34 (2.01, 2.74)	191	27	2.03 (1.21, 3.42)	128	14	0.77 (0.43, 1.38)
≥ 3 ACEs	1461	372	4.13 (3.60, 4.74)	116	20	1.72 (0.98, 3.02)	56	12	1.60 (0.81, 3.17)

ACEs = Adverse childhood experiences; DZ = Dizygotic twins; MZ = Monozygotic twins; OR = Odds Ratio; CI = Confidence interval.
Models were adjusted for age and sex;
95% CIs are based on robust SEs, calculated using generalized estimating equations;
[a]Any adult psychiatric disorder;
[b]Exposure discordant twins, the number of individual twins from exposure discordant twin pairs in the analysis;
[c]OR for every additional ACE.

© 2024 Daníelsdóttir HB et al. *JAMA Psychiatry.*

eTable 9. Associations between the number of ACEs and any adult psychiatric disorder, additionally including dispensed psychotropic medications as an indication for mild psychiatric disorder not attended by specialist care, in the full cohort and within exposure discordant twin-pairs.

	Model 1 (Full cohort)			Model 2 (Within DZ twins)			Model 3 (Within MZ twins)		
	N total	cases[a]	OR (95% CI)	N[b]	cases	OR (95% CI)	N[b]	cases	OR (95% CI)
**ACE total score**[c]	25,252	6726	1.40 (1.37, 1.44)	4018	1102	1.26 (1.14, 1.38)	2834	882	1.16 (1.03, 1.31)
**Any ACE**									
0 ACE	15,501	3348	1.00 (ref.)	1554	330	1.00 (ref.)	1010	257	1.00 (ref.)
≥ 1 ACE	9751	3378	1.90 (1.80, 2.02)	1554	449	1.49 (1.24, 1.80)	1010	275	1.14 (0.90, 1.46)
**Number of ACEs**									
0 ACE	15,501	3348	1.00 (ref.)	1554	330	1.00 (ref.)	1010	257	1.00 (ref.)
1 ACE	5531	1594	1.48 (1.38, 1.59)	1077	278	1.34 (1.08, 1.65)	724	183	1.06 (0.82, 1.38)
2 ACE	2174	798	2.06 (1.86, 2.27)	301	104	1.66 (1.23, 2.24)	189	55	1.21 (0.82, 1.79)
≥ 3 ACEs	2046	986	3.20 (2.90, 3.53)	176	67	2.20 (1.53, 3.15)	97	37	1.62 (1.04, 2.53)

ACEs = Adverse childhood experiences; DZ = Dizygotic twins; MZ = Monozygotic twins; OR = Odds Ratio; CI = Confidence interval.
Models were adjusted for age and sex;
95% CIs are based on robust SEs, calculated using generalized estimating equations;
[a]Any adult psychiatric disorder;
[b]Exposure discordant twins, the number of individual twins from exposure discordant twin pairs in the analysis;
[c]OR for every additional ACE.

© 2024 Daníelsdóttir HB et al. *JAMA Psychiatry.*

eTable 10. Associations between the number of ACEs and any adult psychiatric disorder in the full cohort and within exposure discordant twin-pairs, excluding twins diagnosed with any psychiatric disorder before age 19 years.

	Model 1 (Full cohort)			Model 2 (Within DZ twins)			Model 3 (Within MZ twins)		
	N total	cases[a]	OR (95% CI)	N[b]	cases	OR (95% CI)	N[b]	cases	OR (95% CI)
**ACE total score**[c]	24,743	2201	1.50 (1.46, 1.55)	3824	371	1.24 (1.08, 1.41)	2704	284	1.15 (0.98, 1.36)
**Any ACE**									
0 ACE	15,309	939	1.00 (ref.)	1490	93	1.00 (ref.)	973	76	1.00 (ref.)
≥ 1 ACE	9434	1262	2.33 (2.13, 2.54)	1490	151	1.66 (1.24, 2.21)	973	86	1.19 (0.82, 1.71)
**Number of ACEs**									
0 ACE	15,309	943	1.00 (ref.)	1490	93	1.00 (ref.)	973	76	1.00 (ref.)
1 ACE	5419	541	1.67 (1.50, 1.87)	1036	87	1.43 (1.04, 1.97)	701	56	1.19 (0.80, 1.77)
2 ACE	2111	302	2.45 (2.13, 2.82)	290	38	2.44 (1.53, 3.91)	181	16	0.86 (0.50, 1.47)
≥ 3 ACEs	1904	441	4.25 (3.75, 4.83)	164	26	1.86 (1.13, 3.03)	91	14	1.90 (1.00, 3.63)

ACEs = adverse childhood experiences; DZ = Dizygotic twins; MZ = Monozygotic twins; OR = Odds Ratio; CI = Confidence Interval.
Models were adjusted for age and sex;
95% CIs are based on robust SEs, calculated using generalized estimating equations;
[a]Any adult psychiatric disorder
[b]Exposure discordant twins, the number of individual twins from exposure discordant twin pairs in the analysis;
[c]OR for every additional ACE.

© 2024 Daníelsdóttir HB et al. *JAMA Psychiatry.*

eTable 11. Associations between the number of ACEs and any diagnosed psychiatric disorder in the full cohort and within exposure discordant twin-pairs, excluding twins diagnosed with any psychiatric disorder before answering the web-based survey with the ACE assessment.

	Model 1 (Full cohort)			Model 2 (Within DZ twins)			Model 3 (Within MZ twins)		
	N total	cases[a]	OR (95% CI)	N[b]	cases	OR (95% CI)	N[b]	cases	OR (95% CI)
**ACE total score**[c]	24,067	1449	1.45 (1.40, 1.51)	3,616	233	1.26 (1.07, 1.48)	2,575	159	1.02 (0.83, 1.24)
**Any ACE**									
0 ACE	15,034	637	1.00 (ref.)	1420	62	1.00 (ref.)	930	50	1.00 (ref.)
≥ 1 ACE	9033	812	2.20 (1.97, 2.45)	1420	103	1.59 (1.12, 2.27)	930	49	0.98 (0.63, 1.52)
**Number of ACEs**									
0 ACE	15,034	637	1.00 (ref.)	1420	62	1.00 (ref.)	930	50	1.00 (ref.)
1 ACE	5263	360	1.66 (1.45, 1.89)	1001	65	1.42 (0.97, 2.07)	674	34	1.11 (0.68, 1.80)
2 ACE	2018	192	2.32 (1.96, 2.75)	273	23	2.20 (1.22, 3.97)	172	9	0.62 (0.32, 1.20)
≥ 3 ACEs	1752	260	3.76 (3.22, 4.38)	146	15	1.96 (1.05, 3.65)	83	6	1.08 (0.50, 2.32)

ACEs = adverse childhood experiences; DZ = Dizygotic twins; MZ = Monozygotic twins; OR = Odds Ratio; CI = Confidence Interval.
Models were adjusted for age and sex;
95% CIs are based on robust SEs, calculated using generalized estimating equations;
[a]Any adult psychiatric disorder
[b]Exposure discordant twins, the number of individual twins from exposure discordant twin pairs in the analysis;
[c]OR for every additional ACE.

© 2024 Daníelsdóttir HB et al. *JAMA Psychiatry.*

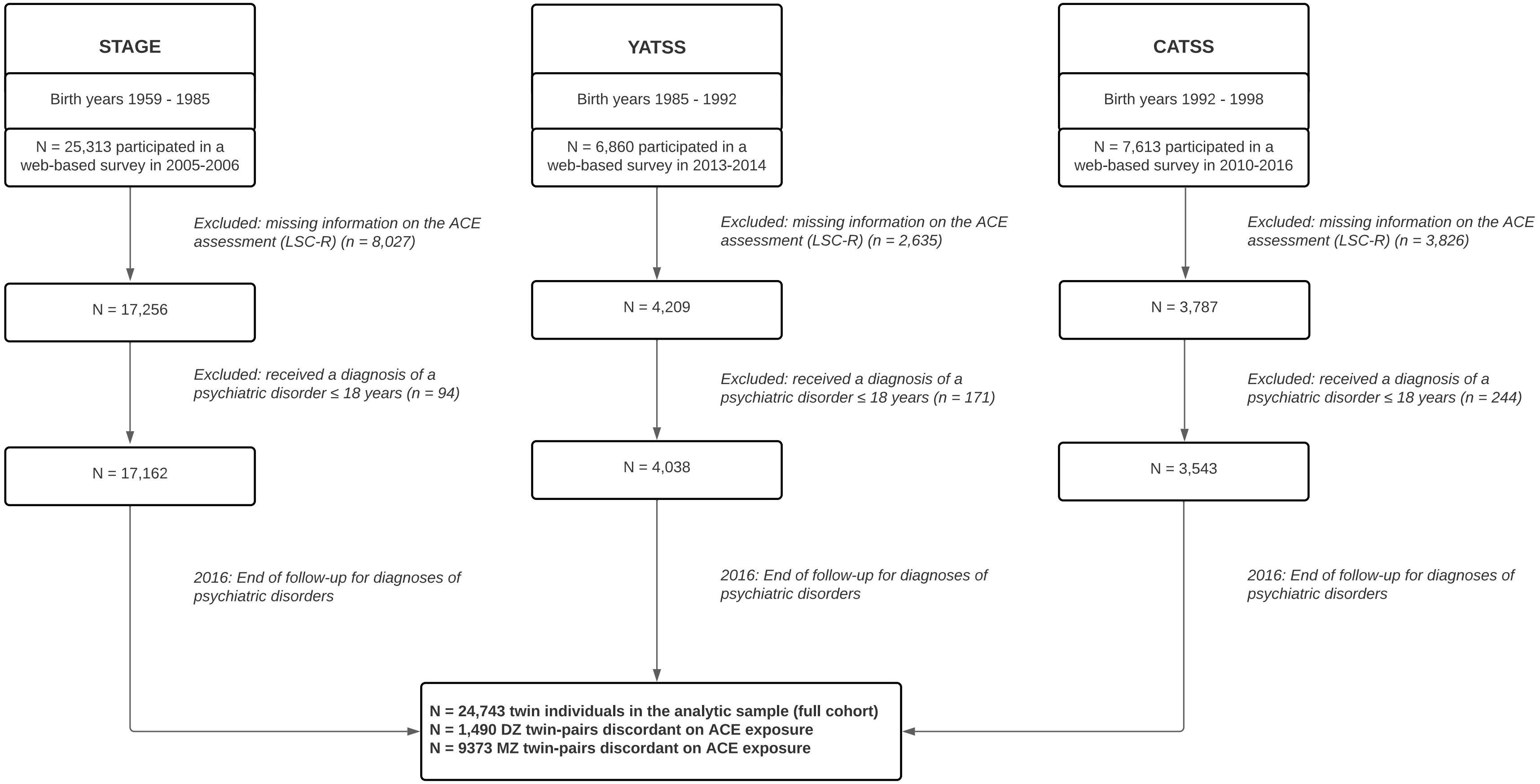

eFigure 1. Flow-chart of the analytic sample excluding twins diagnosed with any psychiatric disorder before age 19 years.

© 2024 Daníelsdóttir HB et al. *JAMA Psychiatry*.

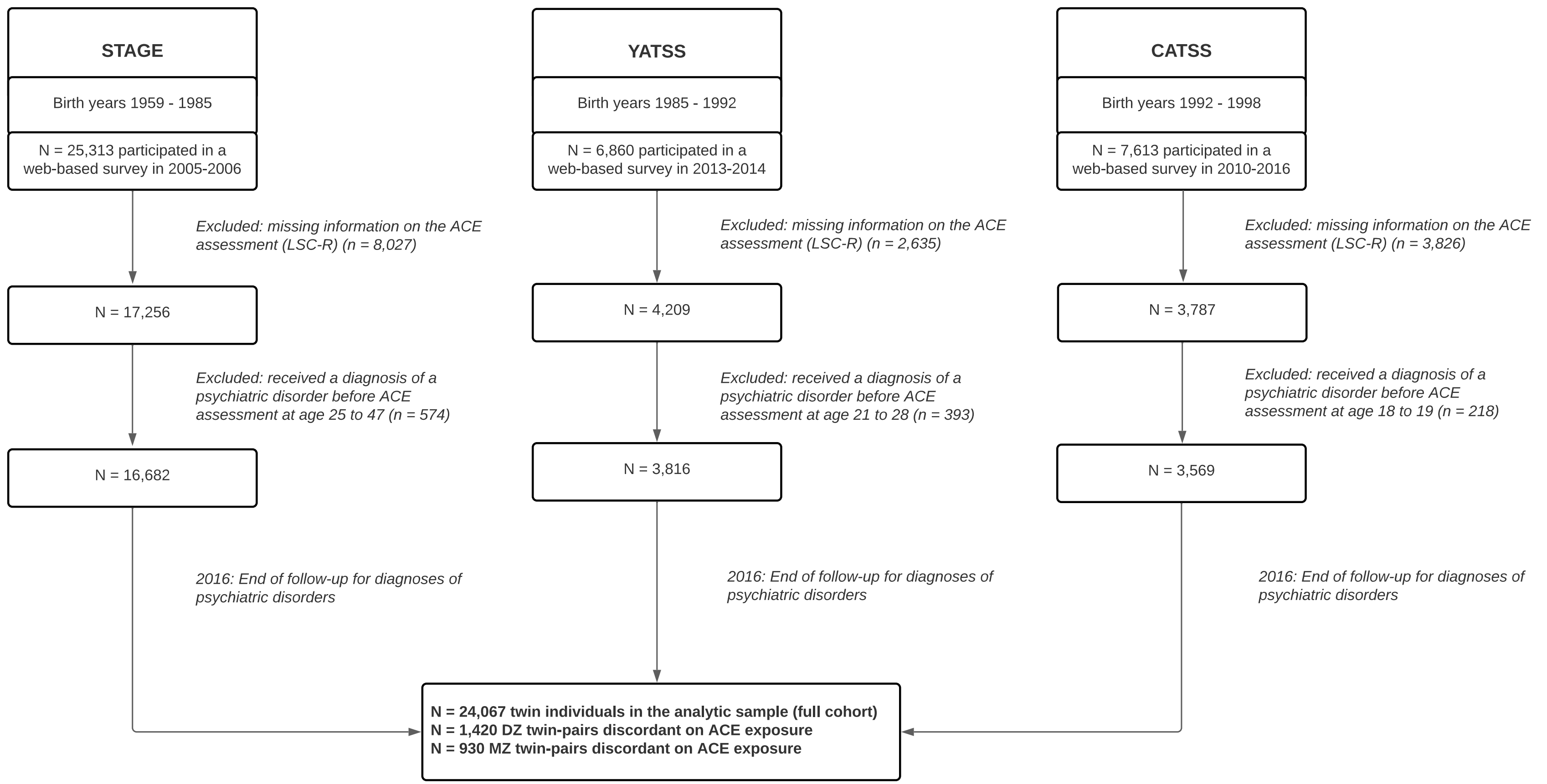

eFigure 2. Flow-chart of the analytic sample where follow-up for psychiatric disorders starts after the web-based surveys with the ACE assessment, excluding twins that received a diagnosis of any psychiatric disorder before answering the survey.

© 2024 Daníelsdóttir HB et al. *JAMA Psychiatry*.

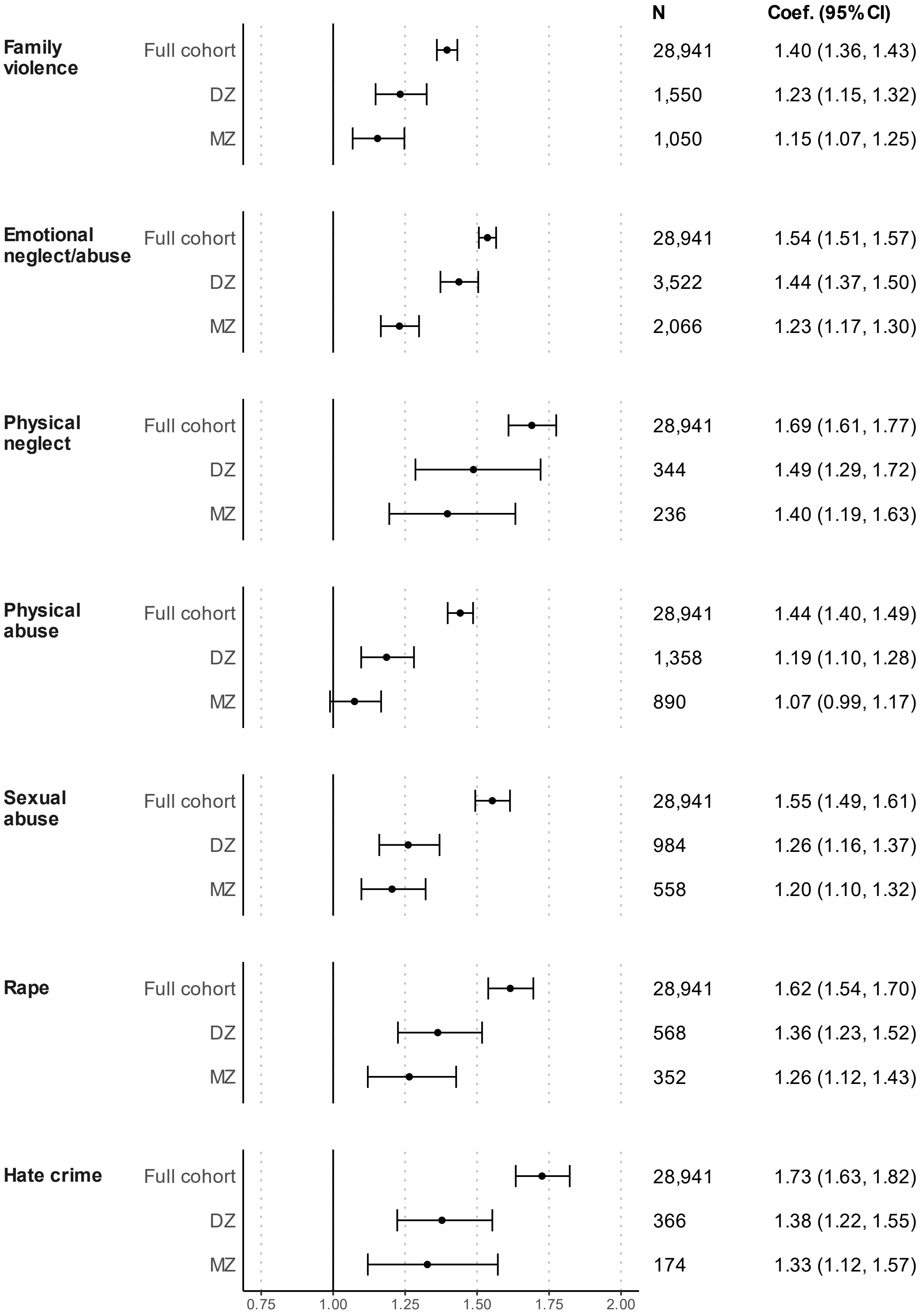

eFigure 3. Associations between ACE subtypes and depression symptoms in the full cohort and within exposure discordant twin-pairs.
Models were adjusted for age and sex; Estimates are given as the percentage increase in depression symptoms, comparing exposed vs. non-exposed twins; 95% CIs are based on

© 2024 Daníelsdóttir HB et al. *JAMA Psychiatry.*

robust SEs, calculated using generalized estimating equations; DZ = Dizygotic twins; MZ = Monozygotic twins; CI = Confidence Interval

© 2024 Daníelsdóttir HB et al. *JAMA Psychiatry.*
