# Supplementary material for: Adverse Childhood Experiences and Adult Mental Health Outcomes
Source: JAMA Psychiatry. 2024 Mar 6;81(6):586–94. doi: 10.1001/jamapsychiatry.2024.0039 (PMC10918580; doi:10.1001/jamapsychiatry.2024.0039)
Supplement: Supplement 2. — Data Sharing Statement [file jamapsychiatry-e240039-s002.pdf]

Data Sharing Statement

Daníelsdóttir. Adverse Childhood Experiences and Adult Mental Health Outcomes. *JAMA Psychiatry*. Published online March 6, 2024. doi:10.1001/jamapsychiatry.2024.0039

Data
Data available: No

Additional Information
Explanation for why data not available: The data used in this study are compiled in the Swedish Twin Registry (STR). We cannot make the data publicly available because of Swedish laws regarding data protection and the ethical approval of the current study. The authors of the present study submitted a research proposal to the STR data management board and the regional ethical review board in Stockholm and got access only to de-identified data, that cannot be shared in any way. However, interested researchers can obtain access to de-identified data by applying to the STR data management board and obtaining an ethical approval from a regional ethical review board (for further information, see https://ki.se/en/research/the-swedish-twin-registry).
